# An In-Vitro Comparison of Shear Bond Strength and Adhesive Remnant Score Between Two Color Change Adhesives in Orthodontic Bonding With Reduced Curing Time Using Different High-Intensity Light Emitting Diode Units

**DOI:** 10.7759/cureus.40951

**Published:** 2023-06-25

**Authors:** Dasari Guravaiah, Konni Prudhvi, Soorabathula Sonika Mani Kiran, Kanaparthy Sri Kavya, RSVM Raghu Ram

**Affiliations:** 1 Orthodontics and Dentofacial Orthopaedics, GSL Dental College & Hospital, Rajahmundry, IND

**Keywords:** high-intensity led, color change adhesive, ari score, reduced curing time, shear bond strength, led curing unit

## Abstract

Aim and objectives: This study aims to determine the effect on shear bond strength (SBS) and adhesive remnant score between two color change adhesives (CCAs) with reduced curing time using different high-intensity light emitting diode (LED) units.

Materials and methods: A total of 108 human first maxillary premolar teeth were randomly allocated into three principal groups (n = 36) based on the type of adhesives used. The adhesives include two CCAs: Transbond^ ^Plus Color Change Adhesive (3M, St. Paul, MN, USA) and Grengloo (Ormco, Orange, CA, USA), and the conventional tooth-colored adhesive: Transbond XT (3M). Each principal group was further divided into three sub-groups (n = 12 each) based on the curing time and type of high-intensity LED units used for bonding the stainless steel brackets. Woodpecker iLED Light Curing Unit (Guilin Woodpecker Medical Instrument Co., Ltd., Guilin, China) was cured for three and six seconds, and the ELIPAR S10 LED Curing Light (3M) was cured for 20 seconds. Bonding of the brackets was done in a standardized manner following the manufacturers' instructions. All the samples were submerged in distilled water at 37^0^C for 24 hours. SBS testing was performed using an Instron machine, and adhesive residue on the debonded surface was examined and scored using a stereomicroscope. Statistical analysis was conducted using one-way ANOVA and Tukey’s post-hoc test.

Results: The results showed significant differences in SBS based on curing time and the type of adhesive resin used. The six seconds curing group exhibited the higher SBS values (15.5 - 22.82 Megapascals [MPa]) followed by the 20 seconds (12.17 - 18.14 MPa) and three seconds (11.31 - 11.74 MPa) groups. Grengloo adhesive demonstrated the highest SBS values among the three types of adhesives. The predominant adhesive remnant scores were 2 and 3.

Conclusions: Grengloo adhesive demonstrates superior bond strength compared to Transbond Plus and Transbond XT. Both Transbond Plus and Grengloo adhesives experience bond failure within the adhesive layer, regardless of the curing intensity or time.

## Introduction

Orthodontic bonding adhesives have evolved from chemically cured composites to color change adhesives (CCAs), which offer advantages such as easy identification of excessive flash and reduced risk to teeth [1]. Conventional adhesive color makes it difficult to distinguish the adhesive-enamel interface during debonding, leading to incomplete adhesive removal and potential enamel loss of 5-150 µm during flash clean-up [2]. CCAs facilitate the removal of adhesive remnants during bracket seating [3]. Transbond Plus (3M, St. Paul, MN, USA) is a pink CCA for orthodontic bonding. The pink dye in Transbond Plus photobleaches during bracket bonding, facilitating flash removal. At cooler temperatures, it appears blue, and after debonding, remnants are easily identified when cooled with air or water. Grengloo (Ormco, Orange, CA, USA) is a green CCA that becomes transparent at body temperature. Cooling the debonded tooth surface with air or water changes the adhesive back to green, facilitating adhesive remnant removal [4]. The strong bond strength between the bracket base and enamel is essential for bracket stability and resistance to occlusal loads. Factors affecting shear bond strength (SBS) include bracket design, adhesive properties, bonding technique, curing units and clinician experience [5]. Altered SBS values were reported with different intensity light emitting diodes (LEDs) at reduced curing time [2,6]. Considering the role of light intensity, a recent study was conducted using a new high-intensity (3200 mW/cm^2^) LED, which showed a mean SBS of 21.56 MPa with six seconds and 15.79 MPa with three seconds of cure time [7]. The remarkable benefit for clinicians lies in the substantially reduced total exposure time for bracket placement in both dental arches, amounting to just over one minute. This brief exposure time proves to be a tremendous advantage, enhancing efficiency and convenience during the procedure [8]. However, the high-intensity (3200 mW/cm^2^) LED unit used is relatively expensive for every orthodontist to afford it. Recently, an economical and new generation high-intensity (2400 mW/cm^2^) LED unit has been introduced onto the market, promising an adequate SBS at six seconds cure time with conventional adhesives. No studies were done on SBS of CCAs with reduced curing time and high-intensity LEDs (2400 mW/cm^2^) to date. This study focuses on comparing the SBS and adhesive remnant index (ARI) scores between two CCAs with reduced curing time using different high-intensity LED units.

## Materials and methods

Methodology

The research was conducted at GSL Dental College & Hospital in Rajamahendravaram (Rajahmundry), India. Approval for the in-vitro procedure was granted by the Institutional Ethics Committee of GSL Dental College & Hospital, with the assigned approval number being GSLDC/IEC/2020/004. In order to determine the appropriate sample size, a power analysis was carried out. The analysis indicated that a sample size of 108 would yield a statistical significance level (alpha) of 0.05 with 80% power.

A total of 108 human maxillary first premolar teeth were used in this in-vitro study. Standardization procedures were followed to ensure consistency and uniformity among the samples [9,10]. To ensure adherence to standard procedures, all samples underwent processing according to ISO/TS 29022:2013 specifications for bevelled-edge shear bond strength testing. Subsequently, the samples were kept for a maximum of six months in distilled water until bonding. To prepare the tooth surface for bonding, the buccal crown aspect of each tooth was meticulously cleaned. This involved polishing the tooth surface using pumice for a duration of 10 seconds, followed by thorough rinsing with water for an additional 10 seconds. To achieve complete dryness, the tooth surface was then carefully dried for 10 seconds using oil-free compressed air. This step was carried out using a three-way syringe, ensuring the removal of any residual moisture and contaminants. By following these standardized cleaning and drying procedures, the tooth surfaces were effectively prepared, providing an optimal substrate for the subsequent bonding process [6]. The teeth were allocated into three principal groups based on the type of adhesive used: (Group I: Transbond XT (3M) (control); Group II: Transbond Plus Color Change Adhesive (3M); and Group III: Grengloo. Each principal group was further divided into sub-groups (n=12) based on the curing time and LED units used ([Sub-group a: three seconds [Woodpecker iLED Light Curing Unit (Guilin Woodpecker Medical Instrument Co., Guilin, China)]; Sub-group b: six seconds [Woodpecker iLED Light Curing Unit (Guilin Woodpecker Medical Instrument Co.); and Sub-group c: 20 seconds (ELIPAR S10 LED Curing Light [3M]). The teeth roots were then covered in colored self-curing acrylic blocks up to the cementoenamel junction, leaving just the crowns, to aid in identification. The buccal sides of the teeth were positioned so that they were parallel to the block's base. To prepare the buccal surface of each tooth, etchants (3M ESPE Scotchbond Etchant; 3M) and primers (Transbond XT Light Cure Adhesive; 3M) were applied to all samples as directed by the manufacturer [11]. The base of the maxillary first premolar pre-adjusted edgewise stainless-steel brackets (Unitek Gemini Twin Brackets; 3M) were coated with the appropriate adhesives, pressed firmly on the mid of the buccal surfaces of the teeth, and the flash was removed with a probe [12].

In this in-vitro setup, the adhesive was cured using different exposure times depending on the specific sub-group being investigated. For sub-group ‘a’, a single exposure of approximately three seconds was applied to the distal side of the bracket. In sub-group ‘b’, the adhesive was cured for a total of six seconds, with three seconds of exposure on both the distal and mesial sides of the bracket. Lastly, for sub-group ‘c’, a total of 20 seconds of curing time was used, with a 10 seconds exposure on both the distal and mesial sides of the bracket. Following the curing process, all samples were incubated in distilled water at 37^0^C for 24 hours. This incubation period simulated oral conditions and allowed for the stabilisation of the adhesive bond. A universal testing machine (UTM) (Instron, Norwood, MA, USA) outfitted with a 1kN load cell was used to evaluate the SBS. The sample was securely locked onto the lower jaw compartment of the UTM, while the upper jaw compartment held a sharp rod. The rod was positioned in such a way that it incised the area between the base and wings of the bracket attached to the teeth when stress was applied in an occluso-gingival manner. The force required for orthodontic bracket debonding was recorded in Newtons (N). The force was then divided by the bracket pad's surface area to determine the SBS values. This allowed for the determination of the force per unit area, which was expressed in Megapascals (MPa). This standardized measurement provided a quantitative assessment of the bond strength for each individual sample.

Evaluation of surface characteristics

The debonded enamel surfaces were carefully examined using a light stereomicroscope (Infinity IOX-6745 Stereo Zoom Microscope; I7 Opto Electronics Inc., Ambala, Haryana, India) at a magnification of 20x. This allowed for a detailed evaluation of the mode of failure. The images captured during the examination were saved to a desktop computer for further analysis. The proportion of adhesive that remained on the tooth surface after bracket removal was then evaluated and given a score by the modified ARI. The modified ARI scores of the three groups were tabulated using the findings of the SBS tests.

By means of IBM version 20 SPSS software (IBM Corp., Armonk, NY, USA), the acquired data was examined. The data was analysed using an ANOVA with Kruskal-Wallis and post-hoc Mann-Whitney U tests for multiple pairwise comparisons. P≤0.05 was studied as statistically significant.

## Results

Statistically significant differences were seen in the SBS values between the three groups (P<0.001). The highest mean values of SBS were seen with six-second curing time in each of the three types of adhesives. These differences among the groups were statistically significant (Table 1).

**Table 1:** Comparison of shear bond strength based on curing time in each of the three adhesives. One way analysis of variance; * denotes significance; p≤0.05 considered statistically significant

Adhesive	Curing time (second)	Curing Unit and Intensity	n	Mean	Standard Deviation	P value
Transbond XT (control)	20	ELIPAR S10 LED Curing Light 1400 mW/cm^2^	12	12.175000	1.9316197	0.001*
	6	Woodpecker iLED Light Curing Unit 2400 mW/cm^2^	12	15.558333	2.6471797	
	3	Woodpecker iLED Light Curing Unit 2400 mW/cm^2^	12	11.745000	2.3677319	
Transbond Plus	20	ELIPAR S10 LED Curing Light 1400 mW/cm^2^	12	15.908333	5.0574353	<0.001*
	6	Woodpecker iLED Light Curing Unit 2400 mW/cm^2^	12	20.996667	4.3961395	
	3	Woodpecker iLED Light Curing Unit 2400 mW/cm^2^	12	11.319167	3.2411459	
Grengloo	20	ELIPAR^TM^ S10 LED Curing Light 1400 mW/cm^2^	12	18.143333	3.8989727	<0.001*
	6	Woodpecker iLED Light Curing Unit 2400 mW/cm^2^	12	22.820833	4.3212823	
	3	Woodpecker iLED Light Curing Unit 2400 mW/cm^2^	12	11.370000	2.3089510	

The highest mean SBS values were observed with Grengloo adhesive for the six seconds with high-intensity LED and the 20 seconds curing times with ELIPAR S10 LED Curing Light and these differences were statistically significant. With three seconds of curing time, high-intensity LED Transbond XT showed the highest mean SBS values compared to the other two CCAs; however, these differences were not statistically significant (Table 2).

**Table 2:** Comparison of shear bond strength based on the type of adhesive in each of the three curing times. ANOVA; p≤0.05 considered statistically significant; * denotes significance

Curing time (second)	Curing Unit and Intensity	Adhesive	Sample Size	Mean	Standard Deviation	P value
20	ELIPAR S10 LED Curing Light 1400 mW/cm^2^	Transbond XT	12	12.175000	1.9316197	0.002*
		Transbond Plus	12	15.908333	5.0574353	
		Grengloo	12	18.143333	3.8989727	
6	Woodpecker iLED Light Curing Unit 2400 mW/cm^2^	Transbond XT	12	15.558333	2.6471797	<0.001*
		Transbond Plus	12	20.996667	4.3961395	
		Grengloo	12	22.820833	4.3212823	
3	Woodpecker iLED Light Curing Unit 2400 mW/cm^2^	Transbond XT	12	11.745000	2.3677319	0.913
		Transbond Plus	12	11.319167	3.2411459	
		Grengloo	12	11.370000	2.3089510	

An inter-group comparison was not done because there was no statistically significant difference in the six seconds group. For 20 seconds of curing time with ELIPAR S10 LED Curing Light, Grengloo adhesive demonstrated a statistically significant difference with Transbond XT. Grengloo and Transbond Plus demonstrated significantly greater SBS than Transbond XT for the six seconds curing time with high-intensity LED. However, the Grengloo and Transbond Plus adhesives did not significantly differ from one another (Table 3).

**Table 3:** Multiple pairwise comparisons of shear bond strength based on the type of adhesive at 20 and six seconds curing time. Tukey’s post hoc tests; * denotes significance

Curing time (second)	Curing Unit and Intensity	Reference Group	Comparison Group	Mean Difference	P value
20	ELIPAR S10 LED Curing Light 1400 mW/cm^2^	Transbond XT	Transbond Plus	-3.7333333	.060
			Grengloo	-5.9683333^*^	.002
		Transbond Plus	Transbond XT	3.7333333	.060
			Grengloo	-2.2350000	.342
		Grengloo	Transbond XT	5.9683333^*^	.002
			Transbond Plus	2.2350000	.342
6	Woodpecker iLED Light Curing Unit 2400 mW/cm^2^	Transbond XT	Transbond Plus	-5.4383333^*^	.004
			Grengloo	-7.2625000^*^	.000
		Transbond Plus	Transbond XT	5.4383333^*^	.004
			Grengloo	-1.8241667	.489
		Grengloo	Transbond XT	7.2625000^*^	.000
			Transbond Plus	1.8241667	.489

The modified ARI frequency distribution analysis revealed differences in adhesive remnants among the study groups. Variations were observed based on the curing units and intensities, as well as the curing times. The Transbond XT control group consistently exhibited bond failure at the adhesive interface at six (intensity of 2400 mW/cm^2^) and 20 seconds (intensity of 1400 mW/cm^2^). However, at three seconds with the intensity of 2400 mW/cm^2^ bond failure is at bracket mesh. Transbond Plus and Grengloo exhibited a generalized distribution of ARI scores at different curing times and intensities. These findings highlight the influence of curing parameters on the effectiveness of adhesive bonding, suggesting that specific combinations of curing units, intensities, and times may impact the quality of orthodontic adhesive bonding (Table 4).

**Table 4:** Modified adhesive remnant index (ARI) frequency distribution between the study groups. Modified ARI: score 0 = no adhesive left on the tooth; score 1 = 1%-25% of adhesive left on the tooth; score 2 = 26%-50% adhesive left on the tooth; score 3 = 51%-75% adhesive left on the tooth; score 4 = 76%-99% adhesive left on the tooth; score 5 = all adhesive left on the tooth with a distinct impression of the bracket mesh

Composite	Curing Unit and Intensity	Curing time (second)	Modified ARI Score					
			0	1	2	3	4	5
Transbond XT (Control)	Woodpecker iLED Light Curing Unit 2400 mW/cm^2^	3	1	1	2	2	2	4
		6	2	2	4	3	1	0
	ELIPAR S10 LED Curing Light 1400 mW/cm^2^	20	1	3	2	4	1	1
Transbond Plus	Woodpecker iLED Light Curing Unit 2400 mW/cm^2^	3	1	1	2	3	2	3
		6	3	3	1	2	2	1
	ELIPAR S10 LED Curing Light 1400 mW/cm^2^	20	1	3	2	3	3	0
Grengloo	Woodpecker iLED Light Curing Unit 2400 mW/cm^2^	3	2	0	3	2	3	2
		6	2	3	4	3	0	2
	ELIPAR S10 LED Curing Light 1400 mW/cm^2^	20	1	2	3	1	2	1

## Discussion

CCAs were developed and introduced to the orthodontic market with the aim of improving the differentiation between adhesive and enamel. The effectiveness of CCAs relies on the polymerization process, which is directly influenced by the total light energy absorbed by the CCA resin. This polymerization process is similar for both CCAs and conventional resins and plays a crucial role in achieving sufficient strength of the cohesive composite resin when using regular LEDs. The total light energy is calculated by multiplying the intensity of the light by the exposure time. When the total light energy is higher, it contributes to an increase in the fracture toughness and flexural strength of the resin material. This enhanced strength is subsequently transferred to the orthodontic brackets that are bonded to the teeth, ultimately resulting in higher shear bond SBS [13]. No literature was available regarding the influence of high-intensity LEDs on the curing of CCAs. Rapid polymerization is a benefit of high-intensity curing units, but they also cause significant polymerization stresses, which degrade the attachment to tooth structure [14]. However, in bracket bonding, the adhesive is thinner than 2 mm, and the bond is created on the tooth's outside, which lessens the possibility of polymerization pressures [15].

Grengloo exhibited the highest mean SBS compared to Transbond Plus and Transbond XT at 20 seconds. This finding suggests that Grengloo might offer superior bonding properties in terms of shear strength when exposed to a curing unit with 1400 mW/cm^2^ intensity. However, it is important to note that the statistical significance of this result was not explicitly mentioned, which may affect the confidence in the observed differences. Grengloo again demonstrated the highest mean SBS with a shorter curing time of six seconds. Later, Transbond Plus displayed a higher SBS than Transbond XT. These findings suggest that Grengloo provides better bonding performance even under shorter curing times and at a high intensity (2400 mW/cm^2^). Similar to the current study, higher SBS like 21.56 MPa and 33 MPa were reported on curing for six seconds at increased intensities of 3200 mW/cm^2^ [4] and at 5000-6000 mW/cm^2^ [16], respectively. This change in the bond strength is directly proportional to the intensity of the curing unit. Interestingly, at a significantly shorter curing time of only three seconds with the same Woodpecker iLED Light Curing Unit, none of the three adhesives showed a statistically significant difference in SBS. This result implies that at this particular curing time, all three adhesives performed similarly in terms of SBS. But according to Reynolds, a minimum time of three seconds generated SBS values strong enough to withstand the forces generated by both orthodontic forces and mastication [17]. Similar to the present study, but with a little more high-intensity (3200 mW/cm^2^) LED unit on CCAs at three seconds produced a distinct SBS value of 15.79 MPa [4], indicating that with an increase in intensity levels, the SBS increases irrespective of the type of CCAs. Nonetheless, all experimental and control groups produced bond strengths that can withstand normal orthodontic forces. However, curing for three and six seconds should be done with caution, due to reduced time duration. The adhesive used to bond brackets must be sufficiently strong to keep the bracket in place during the course of the treatment while still allowing for safe debonding. On the other hand, extremely high values of SBS may raise the chance of enamel breaking during debonding. The most secure method to prevent enamel fracturing during debonding may be an adhesive failure at the composite bracket interface [4].

In the current study, a predominant modified ARI score of 2 followed by 3 was reported among the groups. Due to the limited exposure to light, enamel adhesive failure could be an indication of insufficient resin polymerization at the bracket's base [18]. However, this could reduce the likelihood that the enamel will be harmed during debonding, which is desirable. In the current investigation, ARI scores of group Transbond XT with three seconds curing time (Woodpecker iLED Light Curing Unit) were found to be substantially higher (score 5) than six seconds and 20 seconds. This indicates a lower adhesive capacity and less adherence to the bracket base, this results in a higher ARI score [19]. The samples subjected to the Instron were revealed to have five fractures, four of them were cured for six seconds using a Woodpecker iLED Light Curing Unit, with 20.53 MPa - 22.45 MPa SBS value and one for 20 seconds using an ELIPARTM S10 LED Curing Light, with 18.33 MPa SBS value.

Limitations

Direct comparisons to clinical performance may not be feasible due to the inherent limitations of the in-vitro investigation. The experimental conditions utilised in the laboratory setting may introduce variations in the measured SBS values. Furthermore, it is worth considering that debonding forces of brackets can differ between in-vivo and in-vitro environments. A further point worth mentioning is that this study did not evaluate the potential rise in pulpal temperature linked to high-intensity LED units.

Further scope

Further research is required to evaluate the clinical ease of removal of flash, the bond failure rate of brackets, and the presence of adhesive remnants when using CCAs with high-intensity LED units. Additionally, it is important to investigate the potential rate of pulpal and enamel damage associated with the use of CCAs in conjunction with high-intensity LED units. These areas require in-depth research to better understand the effects and ensure patient safety during orthodontic treatments.

## Conclusions

Grengloo adhesive demonstrates superior bond strength compared to Transbond Plus and Transbond XT. Higher curing unit intensities, such as the Woodpecker iLED light curing unit with an intensity of 2400 mW/cm^2^ enhance the performance of Grengloo adhesive. A curing time of six seconds with Grengloo adhesive yields better bonding results compared to three and 20 seconds. Both Transbond Plus and Grengloo adhesives exhibit bond failure within the adhesive layer, regardless of the curing intensity or time.
